# Predictors of surgical difficulty in upper third molar removal: a prospective cohort study

**DOI:** 10.4317/medoral.26313

**Published:** 2023-12-27

**Authors:** Alba Sánchez-Torres, Anaïs Paños-Crespo, Miquel Sales-Collado, Karla Fuentes-Cazar, Rui Figueiredo, Eduard Valmaseda-Castellón, Cosme Gay-Escoda

**Affiliations:** 1 DDS, MS, PhD. Associate professor of Oral Surgery, Faculty of Medicine and Health Sciences, University of Barcelona (Spain). Researcher of the IDIBELL Institute, Barcelona, Spain; 2 DDS, MS. Master degree program in Oral Surgery and Implantology, Faculty of Medicine and Health Sciences, University of Barcelona, Spain; 3 DDS, MS, PhD. Professor of Oral Surgery, Faculty of Medicine and Health Sciences, University of Barcelona. Researcher of the IDIBELL Institute, Barcelona, Spain; 4 DDS, MS, PhD, EBOS. Chairman and Professor of the Oral Surgery Department, Faculty of Medicine and Health Sciences, University of Barcelona (Spain). Coordinator and Researcher of the IDIBELL Institute, Barcelona, Spain; 5 MD, DDS, MS, PhD, EBOS, OMFS. Former Chairman and Professor of Surgical, Oral and Maxillofacial Pathology, Faculty of Medicine and Health Sciences, University of Barcelona (Spain). Director of the Master degree program in Oral Surgery and Implantology (EFHRE International University/FUCSO). Researcher of the IDIBELL Institute, Barcelona (Spain). Head of the Department of Oral Surgery, Oral Implantology and Maxillofacial Surgery, Teknon Medical Center, Barcelona, Spain

## Abstract

**Background:**

Upper third molar (U3M) removal is a common surgical procedure. The aims of this study were to assess the patient-specific, radiological and surgical factors related to the difficulty of U3M removal, and to determine the incidence of intraoperative and postoperative complications.

**Material and Methods:**

A prospective cohort study was carried out in adult patients undergoing U3M removal. Operative time, surgeon-reported difficulty and the Parant classification were used to assess extraction difficulty. Clinical, radiological and surgical factors were recorded to determine their relationship with surgical difficulty. A descriptive, bivariate and multivariate statistical analysis was carried out.

**Results:**

A total of 250 patients were included. The mean operative time was 10.4 (±12.3) minutes, mean surgeon-reported difficulty was 3.2/10 (±2.3). The multivariate analysis showed greater impaction against the second molar and greater soft tissue and bony impaction to significantly increase operative time and surgeon-perceived difficulty. Additionally, surgeon experience was related to perceived difficulty. The overall incidence of intraoperative complications was 0.8%, and no postoperative events were recorded.

**Conclusions:**

Upper third molars in close relation with the roots of the adjacent second molar and with soft tissue and bony impaction are significantly more difficult to extract. Perceived difficulty was related to surgeon experience. This procedure appears to produce few intra- and postoperative complications.

** Key words:**Upper third molar, surgical extraction, surgical difficulty, operative time.

## Introduction

Third molar extraction is one of the most frequent procedures in Oral and Maxillofacial Surgery, and requires exhaustive diagnostic planning, intraoperative skills and postsurgical considerations (1-3). This is why healthcare professionals must base their practice on scientific evidence (4). Not only radiological variables but also other factors influence the degree of surgical difficulty (5,6). For instance, discrepancies between the radiological and the real dental anatomy might increase the surgical difficulty and can force surgeons to change the initially planned surgical technique (7). Traditionally, the incidence of complications associated with upper third molar (U3M) removal has been considered to be lower than in the case of removal of mandibular third molars. Nonetheless, relevant complications such as oroantral communications can still occur (8-12).

Most predictors of U3M surgical difficulty can be detected by thorough preoperative assessment, allowing the surgeon to avoid potential intra- and postoperative complications (13). The Pell and Gregory, Pederson and Winter classifications are commonly used to determine third molar extraction difficulty, even though they are only based on radiological parameters. Also, according to a study by Alvira-Gonzalez *et al*. (14), they seem to lack inter- and intra-examiner sensitivity and reproducibility. For these reasons, the assessment of other clinical and surgical factors should be considered (15-17). In this regard, a new preoperative assessment form was published by Gay-Escoda *et al*. (18) to help clinicians decide an appropriate treatment plan or a referral decision.

Gender, age and body mass index (BMI) are some of the most widely studied clinical variables. According to Carvalho *et al*. (19), women may be at higher risk of complications. On the other hand, older age has been associated with more complex extractions (7,13). Regarding ethnicity, Renton *et al*. (16) reported that African and Afro-american subjects were predisposed to more complex extractions due to a higher proportion of impacted wisdom teeth. Several clinical variables may play an important role in the surgical difficulty of U3M, so further research on this topic is required.

Surgeon experience is an important factor when assessing operative time, since less experienced professionals usually require more time to extract third molars (10,17,20).

In general, clinicians find U3M removal to be a straightforward procedure. However, some U3M extractions might be quite complex and difficult to predict beforehand. Thus, it would be of great interest to identify variables that can help predict surgical difficulty. Indeed, this information would be useful to prevent complications, improve the preoperative information given to the patient, and accurately assess the operative time. Moreover, the identification of high risk cases might be of interest in public healthcare systems in order to decide which patients should be treated in specialized care centers. The present study was therefore carried out to assess the patient-specific, radiological and surgical factors related to the difficulty of U3M removal, and secondarily to determine the incidence of intra- and postoperative complications.

## Material and Methods

A prospective cohort study was conducted in patients requiring U3M extraction between October 2020 and October 2022 at the Dental Hospital of the University of Barcelona (L’Hospitalet de Llobregat, Barcelona, Spain). The study was conducted in accordance with the guidelines of the Declaration of Helsinki, and was approved by the Clinical Research Ethics Committee of the Hospital (protocol number 31/2020). All patients signed a specific informed consent form. The study was carried out following the STROBE guidelines (21).

- Participants

The main inclusion criteria were adult patients requiring the removal of an U3M (erupted, semi-erupted or impacted), the absence of relevant systemic disease conditions (American Society of Anesthesiologists (ASA) score I and II), and the absence of associated disease of the adjacent second molar. The following exclusion criteria were established: patients receiving antibiotic prophylaxis or any drug treatment capable of interfering with the healing process, patients with acute pericoronitis or severe periodontal disease, and individuals in whom local anesthetic administration was contraindicated.

If a patient was considered to be a candidate for extraction of both U3M, only one extraction was considered randomizing it by tossing a coin.

- Surgical technique

All procedures were carried out under sterile conditions and were performed by first, second and third year students of the Master degree program in Oral Surgery and Implantology of the University of Barcelona (Barcelona, Spain). Upper third molar removal was performed under local anesthesia using a supraperiosteal technique on the buccal and palatine aspects with 4% articaine with epinephrine 1:100,000 solution (Artinibsa; Inibsa, Llica de Vall, Spain). If necessary, the surgeon raised a triangular full-thickness flap. Soft tissue protection was performed with the Minnesota retractor, and bone removal was carried out with a straight handpiece (40,000 rpm) using a round tungsten carbide bur with abundant saline irrigation. Luxation and tooth removal were carried out with elevators and/or forceps. Finally, after curettage of the socket, suturing was performed using 3/0 polyglactin 910 (Vicryl® - Ethicon, Inc., USA).

After the surgical procedure, antiinflammatory medication (ibuprofen EFG, Normon® 600 mg, one Tablet every 8 hours for two days and, if necessary, for up to 3 days) and 0.12% chlorhexidine rinses (PerioAid; Dentaid®, Cerdanyola del Vallés, Spain, 15 ml every 12 hours for 7 days starting 24 hours postoperatively) were prescribed. Paracetamol (Paracetamol, Normon®, 1 g, one Tablet every 8 hours) was prescribed as rescue analgesia. The patient was advised to contact the center if he/she detected any unusual postoperative signs or symptoms. Postoperative instructions were given verbally and in writing.

- Study variables

The following variables were recorded: (a) Clinical variables (age, gender, BMI, ethnicity and anxiety level according to the Spanish version of the Modified Dental Anxiety Scale (MDAS) (22) and the Dental Fear Survey (DFS) (23)); (b) Radiological variables (Pell and Gregory and Winter classifications, number of roots detected in the panoramic radiograph and after extraction, root morphology (under development, favorable (convergent and/or parallel), unfavorable (divergent, curved, separated, bulbous, dilacerated), and relationship with the second molar and maxillary sinus); and (c) Surgical variables (surgeon experience).

The main outcome variables were operative time (measured in minutes from the beginning of the incision to the end of suturing or to the end of curettage of the socket if sutures were not required), surgical technique (according to the modified Parant classification (24)) and surgeon-reported difficulty based on a visual analogue scale (VAS) from 0-10 cm.

Intraoperative (bleeding, oroantral communication and displacement of the tooth into neighboring anatomical spaces) and postoperative complications (postoperative infection, dry socket, maxillary sinusitis) were also recorded.

- Calibration

Three investigators (APC, MSC and KFC) underwent inter-examiner calibration for the variables of depth, available distal space and angulation (according to the Pell and Gregory and Winter classifications) by analyzing 10 panoramic radiographs from patients who were not included in this study. The resulting Kappa index was 0.8932 for depth, 0.7865 for angulation and 1 for distal space.

- Sample size calculation

Sample size calculation was performed using the Stata/IC 15.1 package (StataCorp LLC, Lakeway Drive, USA). A proportions estimate was made for a finite population of 3000 patients, based on a known proportion of oroantral communications of 2.4% of cases (12). For an absolute precision of ± 2% and 95% confidence level, 210 subjects were seen to be required. The sample was increased to 250 patients to compensate for possible drop-outs.

- Statistical analysis

A descriptive and bivariate analysis of the data was performed using a Student t-test for scale variables, the chi-square test for dichotomous variables or one-way analysis of variance (ANOVA) for the comparison of means in order to relate the different preoperative variables to surgical difficulty measured by operative time, surgical technique and surgeon-reported difficulty. Finally, a multivariate analysis was performed using linear regressions to determine factors related to increased surgical difficulty according to operative time and surgeon-reported difficulty. Statistical significance was considered for *p* < 0.05.

## Results

**Figure 1:**
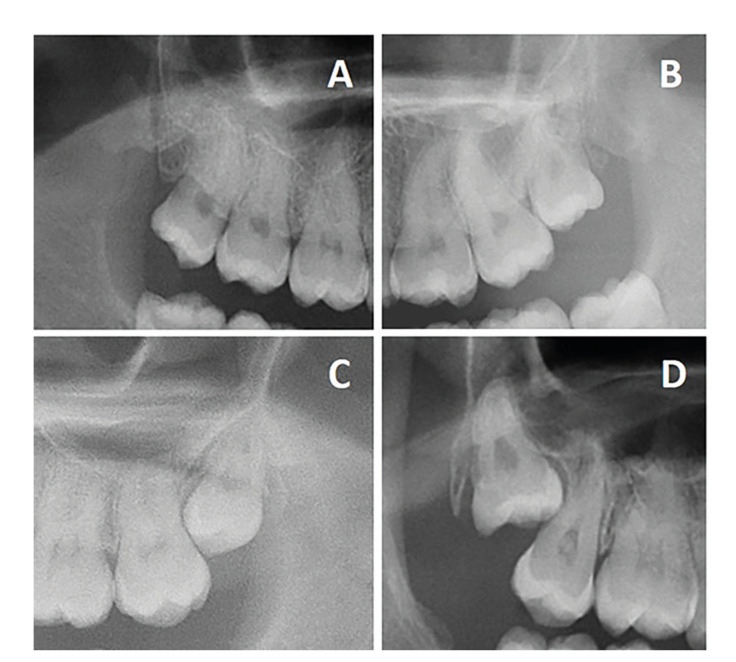
Types of third molar impaction against the second molar. A. Not impacted, B. Crown-impacted, C. Crown and root impacted, D. Root impacted.

A total of 250 patients, 141 females (56%) and 109 males (44%), with a mean age of 28.5 (±9.8) years were included in the study. The mean operative time was 10.4 (±12.3) minutes, the mean surgeon-reported difficulty was 3.2/10 (±2.3) mm, and 214 interventions (86%) were classified as corresponding to type 1 according to the Parant classification (24) (i.e., extraction with forceps and elevators). Table 1 shows the main features of the participants and the bivariate analysis for operative time, surgeon-reported difficulty and surgical technique. None of the clinical variables were related to surgical difficulty. On the other hand, an increase of the following variables were significantly associated to greater difficulty, i.e., soft tissue and bony impaction (especially semierupted and total bony impaction), angulation (mesial and horizontal), greater depth (C) and available distal space, greater impaction against the second molar (mainly root impaction), and proximity to the maxillary sinus (predominantly overlapping and antral roots). In addition, surgical experience showed a significant negative correlation (*p*=0.020) to operative time (i.e., the more experienced the surgeon, the shorter the operative time). Fig. 1 shows the distinct types of third molar impaction against the second molar.

**Table 1:**
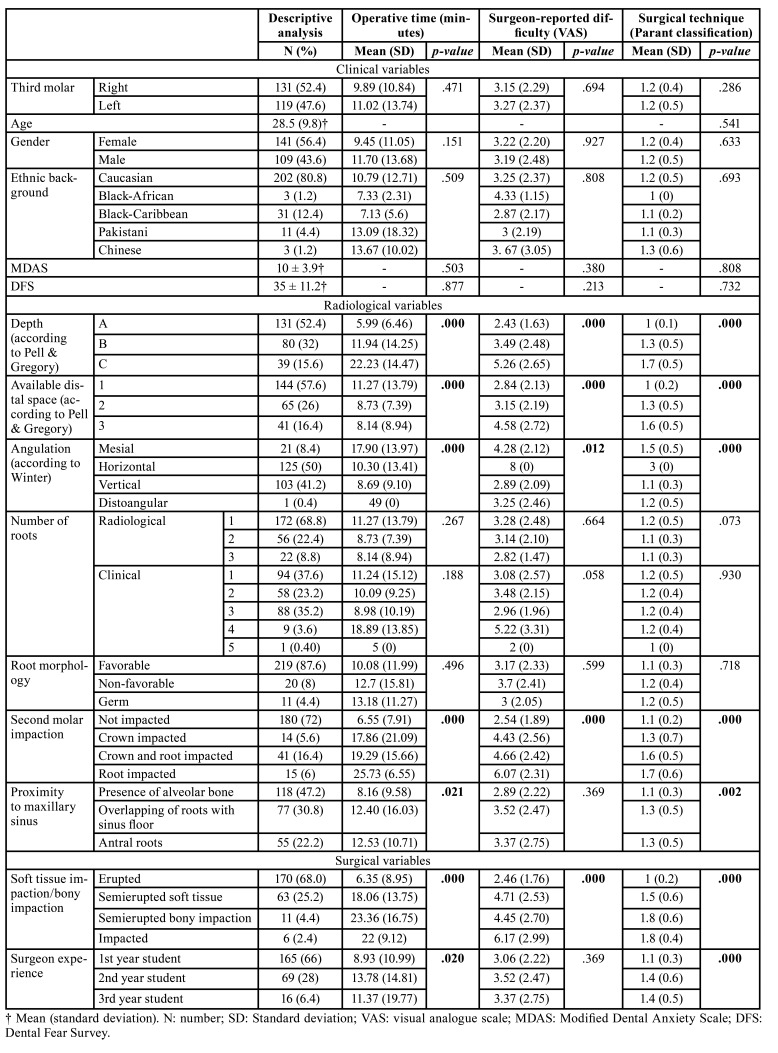
Descriptive and bivariate analysis according to clinical, radiological and surgical characteristics and outcome variables that assess difficulty.

The multivariate analysis (Table 2) showed that greater impaction against the second molar and the need to perform a more complex surgical technique significantly increased operative time and surgeon-perceived difficulty. Additionally, surgeon experience was related to operator-perceived difficulty (*p*=0.017).

One case of bleeding (0.4%) and one oroantral communication (0.4%) were recorded. Both cases were solved with compression and an intraalveolar collagen dressing and followed up until one week with no sequelae. There were no other postoperative complications.

**Table 2:**
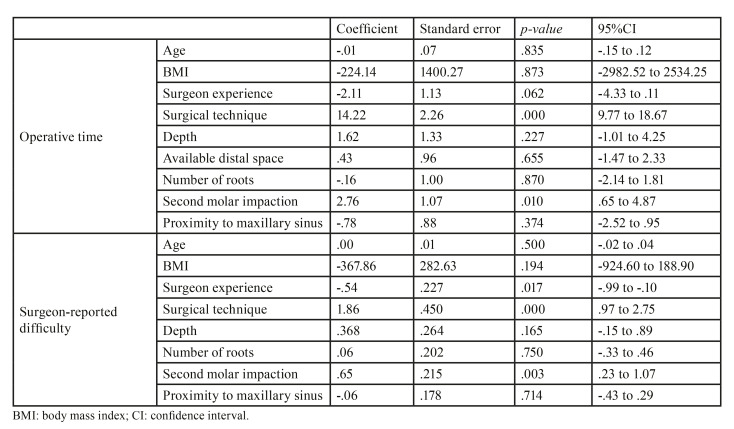
Multivariate linear regression analysis of operative time and surgeon-reported difficulty.

## Discussion

The main findings of the present study are that radiological variables, especially second molar impaction, are significantly related to surgical difficulty of U3M removal. In addition, surgeon experience seems to play an important role in determining operative time and operator-perceived difficulty. In this sample, the clinical variables did not seem to influence surgical difficulty. This outcome is probably related with the fact that most U3M extractions are straightforward and fast, reducing the effect of patient-dependent variables such as age or degree of anxiety. Nonetheless, the removal of some U3M can pose a high degree of difficulty. Thus, it would be of great interest to clinicians to identify which preoperative variables can be associated with such cases.

Although some studies have observed that age (5,14,16,17) or degree of anxiety (2,23,25) are related to the surgical difficulty of mandibular third molar extraction, it seems that these clinical variables have less influence in the case of the extraction of maxillary third molars (18). Indeed, the few available studies that have evaluated the difficulty of U3M removal (17,19,26,27) seem to evidence a relationship between difficulty and surgical technique, surgical experience and anatomical variables such as depth of the U3M, impaction against the second molar and proximity to the maxillary sinus.

The present study was carried out in a university setting in which students of a Master degree program performed all the surgical procedures. Thus, the external validity of the results might be inferior than if experienced clinicians had been involved, since the complexity of the extractions might have been overestimated. Indeed, surgical experience seems to play an important role, since significant differences were found between first, second and third year students. Likewise, Susarla *et al*. (17) analyzed extraction difficulty and concluded that resident experience was related to operative time. In fact, the mean operative time according to Susarla *et al*. (17) was 6.4 (±7) minutes, while in the present study the time was 10.4 (± 12.3) minutes, probably due to the fact that two-thirds of the extractions were performed by first year students.

Factors related to the position of the U3M, specifically depth, angulation and impaction against the second molar, seem to increase difficulty, as observed in other studies (5,19). Similarly, proximity to the maxillary sinus increases the difficulty, as it may require modification of the surgical technique in order to avoid complications (5). In contrast, the lack of distal space does not seem to increase difficulty, probably due to the elasticity of the maxillary tuberosity (19). Indeed, the posterior area of the maxilla has a high proportion of cancellous bone, which is softer and easier to expand during extraction maneuvers in comparison with the mandible (17).

Upper third molar extractions are commonly perceived by clinicians as simple procedures, which may lead to underestimation of the complications - particularly when novice professionals are involved (19). Nevertheless, the present study recorded a low prevalence of intra- and postoperative complications, which seems consistent with the overall low difficulty. However, Pourmand *et al*. (12), in a retrospective study, reported an intraoperative complications rate of 5.1%, of which 2.4% corresponded to oroantral communications. Likewise, Chuang *et al*. (13) recorded a total complications rate of 18.3%, of which 3.9% occurred during surgery and 16.3% in the postoperative period. This study found the most influential factors for the occurrence of complications to be age (specifically patients between 25-35 years of age), a complex anatomical position of the third molar, the presence of a lesion and periodontal disease.

Future research should focus on comparing difficulty assessments between professionals with different levels of experience and training (for example, general dental practitioners versus oral surgeons), in order to have a more complete picture of the learning curve.

## Conclusions

Upper third molars in close relation with the roots of the adjacent second molar and with soft tissue and bony impaction are significantly more difficult to extract. Perceived difficulty is related to surgeon experience. This procedure appears to produce few intra- and postoperative complications.

## References

[1] Akadiri OA, Obiechina AE, Arotiba JT, Fasola AO (2008). Relative impact of patient characteristics and radiographic variables on the difficulty of removing impacted mandibular third molars. J Contemp Dent Pract.

[2] Aznar-Arasa L, Figueiredo R, Valmaseda-Castellón E, Gay-Escoda C (2014). Patient anxiety and surgical difficulty in impacted lower third molar extractions: A prospective cohort study. Int J Oral Maxillofac Surg.

[3] Barreiro-Torres J, Diniz-Freitas M, Lago-Méndez L, Gude-Sampedro F, Gándara-Rey JM, García-García A (2010). Evaluation of the surgical difficulty in lower third molar extraction. Med Oral Patol Oral Cir Bucal.

[4] Farish SE, Bouloux GF (2007). General technique of third molar removal. Oral Maxillofac Surg Clin North Am.

[5] Sánchez-Torres A, Soler-Capdevila J, Ustrell-Barral M, Gay-Escoda C (2019). Patient, radiological, and operative factors associated with surgical difficulty in the extraction of third molars: a systematic review. Int J Oral Maxillofac Surg.

[6] Gbotolorun OM, Arotiba GT, Ladeinde AL (2007). Assessment of factors associated with surgical difficulty in impacted mandibular third molar extraction. J Oral Maxillofac Surg.

[7] Chen SK, Huang GF, Cheng SJ (2001). The relationship between radiologic interpretation and root tip fracture during tooth extraction performed by junior clinicians. Oral Surg Oral Med Oral Pathol Oral Radiol Endod.

[8] del Rey-Santamaría M, Valmaseda Castellón E, Berini Aytés L, Gay Escoda C (2006). Incidence of oral sinus communications in 389 upper thir molar extraction. Med Oral Patol Oral Cir Bucal.

[9] de Santana-Santos T, de Souza-Santos JA, Martins-Filho PR, da Silva LC, de Oliveira e Silva ED, Gomes AC (2013). Prediction of postoperative facial swelling, pain and trismus following third molar surgery based on preoperative variables. Med Oral Patol Oral Cir Bucal.

[10] Phillips C, White RP Jr, Shugars DA, Zhou X (2003). Risk factors associated with prolonged recovery and delayed healing after third molar surgery. J Oral Maxillofac Surg.

[11] Yuasa H, Sugiura M (2004). Clinical postoperative findings after removal of impacted mandibular third molars: prediction of postoperative facial swelling and pain based on preoperative variables. Br J Oral Maxillofac Surg.

[12] Pourmand PP, Sigron GR, Mache B, Stadlinger B, Locher MC (2014). The most common complications after wisdom-tooth removal: part 2: a retrospective study of 1,562 cases in the maxilla. Swiss Dent J.

[13] Chuang SK, Perrott DH, Susarla SM, Dodson TB (2007). Age as a risk factor for third molar surgery complications. J Oral Maxillofac Surg.

[14] Alvira-González J, Figueiredo R, Valmaseda Castellón, Quesada-Gómez C, Gay Escoda C (2017). Predictive factors of difficulty in lower third molar extraction: a prospective cohort study. Med Oral Patol Oral Cir Bucal.

[15] Obimakinde O, Okoje V, Ijarogbe OA, Obimakinde A (2013). Role of patients' demographic characteristics and spatial orientation in predicting operative difficulty of impacted mandibular third molar. Ann Med Health Sci Res.

[16] Renton T, Smeeton N, McGurk M (2001). Factors predictive of difficulty of mandibular third molar surgery. Br Dent J.

[17] Susarla SM, Dodson TB (2013). Predicting third molar surgery operative time: a validated model. J Oral Maxillofac Surg.

[18] Gay-Escoda C, Sánchez-Torres A, Borrás-Ferreres J, Valmaseda-Castellón E (2022). Third molar surgical difficulty scales: systematic review and preoperative assessment form. Med Oral Patol Oral Cir Bucal.

[19] de Carvalho RW, de Araújo Filho RC, do Egito Vasconcelos BC (2013). Assessment of factors associated with surgical difficulty during removal of impacted maxillary third molars. J Oral Maxillofac Surg.

[20] Komerik N, Muglali M, Tas B, Selcuk U (2014). Difficulty of impacted mandibular third molar tooth removal: Predictive ability of senior surgeons and residents. J Oral Maxillofac Surg.

[21] Kwakkenbos L, Imran M, McCall SJ, McCord KA, Fröbert O, Hemkens LG (2021). CONSORT extension for the reporting of randomised controlled trials conducted using cohorts and routinely collected data (CONSORT-ROUTINE): checklist with explanation and elaboration. BMJ.

[22] Humphris GM, Morrison T, Linday SJ (1995). The modified dental anxiety scale: validation and United Kingdom norms. Community Dent Health.

[23] Kleinknecht RA, Garcia LJ, Gledhill LW, Beesley KA, Coldwell SE (2007). Development and validation of the Spanish Interval Scale of Anxiety Response (ISAR). Anesth Prog.

[24] García AG, Sampedro FG, Rey JG, Vila PG, Martin MS (2000). Pell-Gregory classification is unreliable as a predictor of difficulty in extracting impacted lower third molars. Br J Oral Maxillofac Surg.

[25] Coolidge T, Chambers MA, García LJ, Heaton LJ, Coldwell SE (2008). Psychometric properties of Spanish language adult dental fear measures. BMC Oral Health.

[26] Pippi R, Bufacchi J, De Luca S, Pietrantoni A (2023). Are there difficulty variables in maxillary third molar surgery?. A prospective observational cohort study. Minerva Dent Oral Sci.

[27] Susarla SM, Dodson TB (2004). Risk factors for third molar extraction difficulty. J Oral Maxillofac Surg.

